# Ovarian reserve in nigerian women with sickle cell anaemia: a cross- sectional study

**DOI:** 10.1186/s13048-021-00927-5

**Published:** 2021-12-11

**Authors:** Sunusi Rimi Garba, Christian Chigozie Makwe, Vincent Oluseye Osunkalu, Olufunto Olufela Kalejaiye, Adaiah Priscillia Soibi-Harry, Amina Umar Aliyu, Bosede Bukola Afolabi

**Affiliations:** 1 Department of Obstetrics and Gynaecology, Lagos University Teaching Hospital, Idi-Araba Lagos, Nigeria P.M.B. 12003, Surulere, Lagos, Nigeria; 2 Department of Obstetrics and Gynaecology, College of Medicine University of Lagos, Idi-Araba, Lagos, Nigeria; 3 Department of Hematology and Blood Transfusion, College of Medicine University of Lagos, Idi-Araba, Lagos, Nigeria; 4 Department of Medicine, College of Medicine University of Lagos, Lagos, Nigeria; 5 Department of Nursing, Lagos University Teaching Hospital, Idi-Araba Lagos, Lagos, Nigeria

**Keywords:** Sickle cell anaemia, Anti-Mullerian hormone, Ovarian reserve, Diminished ovarian reserve, Ovarian sickling, Hydroxyurea, Haemoglobinopathy, Nigeria

## Abstract

**Abstract:**

**Introduction:**

Sickle cell disease is the most common monogenetic disorder worldwide. There have been reports of endocrine dysfunction and gonadal failure among affected individuals, especially in males. The findings on ovarian reserve and failure in women with sickle anaemia have been inconsistent.

**Aim and objective:**

The aim of this study was to determine and compare the ovarian reserve of Nigerian women with and without sickle cell anaemia attending a University Teaching Hospital.

**Study Design:**

This cross-sectional study was carried out at the Adult Sickle Cell Clinic and the Community Health Clinic of the Lagos University Teaching Hospital.

**Methodology:**

A total of 166 participants who met the selection criteria, were recruited for the study. The study population consisted of two groups of women matched for age: 83 women with HbSS and 83 women with HbAA. The haemoglobin phenotype of each participant was determined on alkaline electrophoresis (pH 8.4) before analysis. Serum Anti-Mullerian Hormone (AMH) was determined using Enzyme-linked immunosorbent assay (ELISA) method (Calbiotech Inc. USA, Catalog no AM448T).

**Results:**

The mean ± SD of serum AMH level in women with HbSS was 3.64 ± 0.65 ng/mL and was lower than that of women with HbAA 7.35 ±1.19 ng/mL (*p* < 0.001). Serum AMH negatively correlated with age in both study groups (HbAA and HbSS). Also, a significant negative correlation was found between serum AMH and BMI in women with HbAA.

**Conclusion:**

The study showed diminished ovarian reserve in women with HbSS when compared to age-matched women with HbAA.

## Introduction

Sickle cell disease (SCD) is an inherited autosomal recessive haemoglobinopathy characterized by chronic haemolytic anaemia [1, 2]. SCD consists of the haemoglobin SS (HbSS) and haemoglobin variant syndromes such as haemoglobin SC (HbSC), haemoglobin SE (HbSE) [3]. Sickle cell anaemia (SCA), also known as HbSS, is the most common and most severe form of SCD [4]. The prevalence of sickle cell disease ranges between 10% to 45% in various parts of sub-Saharan Africa [5, 6]. Globally, over 300,000 children are born annually with SCD and about 70% of the births occur in sub-Saharan Africa [7].

In Nigeria, it is estimated that 24% of the population have sickle cell trait [7], making it the country with the largest population of individuals with sickle cell trait worldwide [7]. According to Mulumba et al, about 150,000 Nigerian children are born with sickle cell disease annually [5], in contrast to 250 new SCD births per annum in the United kingdom (UK) and 2000 in the United states (US) [6, 8]. Globally, the life expectancy of women with sickle cell anaemia has improved over the last four decades due to multiple medical advancements in care [6]. The survival estimates have continued to improve from a median survival of 42-48 years in 1994, to 58 years in 2014 [9]. It is therefore, not unusual nowadays to have patients with SCA who are 60 - 70 years old [10].

The implication of people with SCA living longer is that they are inclined to develop complications of the disease. Endocrine complications such as thyroid and gonadal dysfunction may lead to gonadal failure/insufficiency in males and females with undesirable effects on their reproductive health [2, 5]. Gonadal dysfunction inherent to SCA has been described in adults (especially in males), such as hypogonadism, sperm abnormalities, and erectile dysfunction (ED) [11]. Although the available literature is inconclusive about gonadal failure in females with SCA [10, 11], some reproductive issues that have been reported in females with SCA include delayed puberty and premature menopause [11]. Possible explanation for the endocrine dysfunction in patients with SCA include: increased iron storage secondary to frequent blood transfusions, ischemia due to vaso-occlusive crisis and inflammatory mediators during ischemia [8, 12]. Assessment of ovarian reserve or function is an indicator of the reproductive potential of women. There are various markers available for assessing the ovarian reserve which include: anti-Mullerian hormone (AMH), basal follicle stimulating hormone (FSH) levels, basal oestradiol (E2), and antral follicle count [13, 14]. However, serum AMH is considered a novel marker for the assessment of ovarian reserve [2]. AMH is a gonad specific member ofthe transforming growth factor beta (TGF-β) superfamily with a total weight of 140KDa [15, 16]. It is a dimeric glycoprotein produced by the granulosa cells in the ovary and is usually only seen at the preantral and small antral follicle stages [17]. Serum AMH level is usually determined by the number of basal antral follicles [17], and does not exhibit intracycle variability, therefore it can be measured on any day of the menstrual cycle, unlike other biochemical markers such as serum basal FSH and estradiol [17, 18].

Despite the burden of SCD in Nigeria, there is little or no data on the impact of SCA on the ovarian reserve of reproductive-aged women. The aim of this study was to determine and compare the ovarian reserve in Nigerian women with and without SCA using serum AMH as a marker.

## Materials and methods

This was an analytical cross-sectional study carried out at the Adult Sickle Cell and Community Health Outpatient Clinic of the Lagos University Teaching Hospital (LUTH), Lagos, Nigeria. Ethical approval was obtained before the commencement of the study. A total of 166 eligible women who met the selection criteria were recruited for the study between March 2019 and August 2019. The inclusion criteria included non-pregnant women with confirmed HbAA or HbSS phenotype, aged between 18 years and 45 years, and having regular menstruation at the time of the study. Pregnant or lactating women, women with current or past history of oophorectomy/ovarian surgery, combined oral contraceptive usage, infertility/subfertility, blood transfusion (within the past three months), chemotherapy, and/or radiation therapy were excluded from the study. The study population consisted of two age-matched groups based on the haemoglobin phenotype. A total of 83 women with HbSS and 83 women with HbAA were selected for the study.

After obtaining informed written consent, the subjects’ weight and height were measured using a portable stadiometer with movable headpiece mounted on balanced beam scale (FM -S120 Fullmedia China). Thereafter, five milliliters of venous blood were collected from each participant using sterile needles through venipuncture after disinfection of the antecubital fossa with 70% alcohol. Two milliliters of the blood were transferred into EDTA bottles for haemoglobin electrophoresis at alkaline pH (8.4) using the electrophoresis machine (DY 300, Hunan, China), while the remaining 3ml was collected in Serum Separator Tubes (SST) for AMH assay.

The specimen bottles were labelled with the unique identification number and transported via cold chain to the LUTH Central Research Laboratory. The haemoglobin phenotype of all study participants was confirmed before assessing their ovarian reserve. The specimen for AMH assay was allowed to clot at room temperature for 20 minute and centrifuged at 3500 rpm for 10 minutes using Eppendorf 5415C centrifuge (Eppendorf AG, Germany). The serum was collected in 2 mL sterile cryovials and stored at -80^0^C until analysis.

The serum levels of Anti-Mullerian Hormones (AMH) were determined using Enzyme-linked immunosorbent assay (ELISA) method (Calbiotech Inc. USA, Catalog no AM448T). The lowest detectable limit of AMH in this study is 0.18 ng/mL and the highest detectable limit is 22.00 ng/mL according to the manufacturer’s instruction. Inter-assay precision coefficients of variation were 5.3% and 3.7% for level one and two controls, respectively. The intra-assay coefficients of variation were 4.2% and 3.5% for level one and two controls, respectively.

### Statistical analysis

Data were entered and analyzed using the IBM Statistical Package for Social Sciences (SPSS) Version 23.0. Armonk, NY: IBM Corp. The categorical variables were summarized and presented as frequency distribution tables. The test of normality of continuous variables was performed using the Kolmogorov- Smirnov test. The continuous variables that were normally distributed were presented as mean (± standard deviation), The Student’s independent t- test was used to compare mean of continuous variables such as age, body mass index (BMI), and serum AMH in HbSS and HbAA study participants. Pearson’s correlation coefficient was used to determine the relationship between serum AMH with age and BMI. Simple and multivariable linear regressions were used to assess the predictors of serum AMH using age and BMI of the study participants as covariates. The level of significance was set at *P* < 0.05, power of 0.80, normal distribution, equal sample size in both groups.

## Results

  The mean age (±SD) of HbSS study participants at 27.9 (±7.5) years, did not differ significantly from those of HbAA participants 27.4 (±7.9) years (*p* = 0.959). The mean BMI (±SD) of HbSS participants 20.7 (±3.0) kg/m^2^ was significantly lower than 23.6 (±4.2) kg/m^2^ observed among HbAA participants, as expected (Table 1).

**Table 1:** Socio-demographic characteristics of the study participants

Variable	HbSS	HbAA	X^2^	*P-value*
	*N* = 83	*N* = 83		
	n (%)	n (%)		
**Age (year)**				
<35	63(75.9)	65(78.3)	0.3065	0.959
≥35	20(24.1)	18(21.7)		
**Mean age (SD)**	27.9(±7.5)	27.4(±7.9)	0.39283	0.6950‡
**Occupation**				
Skilled professional	38(45.8)	32(38.6)	3.5660	0.312
Semi-skilled professional	22(26.5)	29(34.9)		
Unskilled professional	19(22.9)	14(16.9)		
Unemployed	4(4.8)	8(9.6)		
**Tribe**				
Yoruba	62(74.7)	58(69.9)	1.6889	0.639
Igbo	10(12.1)	15(18.1)		
Hausa	1(1.1)	2(2.4)		
Other	10(12.1)	8(9.6)		
**Parity**				
0	69(83.1)	64(77.1)	3.0526	0.217
≥1	14(16.9)	19(22.9)		
**Alcohol intake**				
Yes	27(32.5)	28(33.7)	0.0272	0.869
No	56 (67.5)	55(66.3)		
**Cigarette smoking**				
Yes	2 (2.4)	1(1.2)	0.3395	0.560
No **BMI (kg/m**^**2**^**)**	81(97.6)	82(98.8)		
**BMI**				
<25	75(90.4)	50(60.2)	23.094	<0.001*
≥25	8(9.6)	33(39.8)		
**Mean BMI(±SD)** **Hydroxyurea**	20.7(±3.0) 5(6.0) 78(94.6)	23.6(±4.2) - -	.0349 0.665	<0.001*‡ 0.736

‡Student t-test statistic

Table 2 Shows mean distribution of serum AMH levels among study participants. The mean serum AMH levels in the study participants with HbSS phenotype (3.64 ± 0.65 ng/mL) when compared to (7.35 ± 1.19 ng/mL) in study participants with HbAA phenotype was significantly lower (*p* < 0.001). Similarly, significantly lower values of serum AMH (0.15 – 1.14 ng/mL**)** were demonstrated among female participants with HbSS compared to HbAA participants (21.7% vs 10%; χ^2^= 9.963, *p*= 0.007).

**Table 2:** Comparison of serum AMH levels between HbSS and HbAA study participants

AMH categories (ng/mL)	HbSS (N=83) n (%)	HbAA (N=83)		
		n (%)	X^2^	*P-value*
**Low (0.15 – 1.14)**	18 (21.7)	9 (10.8)	9.963	0.007*
**Normal (1.15-2.56)**	14 (16.9)	5 (6.0)		
**High (>2.65)**	51 (61.4)	69 (83.1)		
**Mean (SD)**	3.64 (0.65)	7.35 (1.19)	-5.398‡	<0.001*

‡student’s t test, * Statistically significant

Table 3 shows a negative correlation between serum AMH levels and age in both HbSS (*r*= **-**0.255**;***p* = 0.020) and HbAA study participants (*r*= -0.425; *p* < 0.001). Conversely, while BMI showed a weak but significant negative correlation with serum AMH levels among HbAA subjects (*r* = -0.240, *P*-value = 0.029), no significant correlation was observed between BMI and AMH among HbSS participants (*r* = 0.035, *p*-value = 0.733).

**Table 3:** Correlation analysis of serum AMH and age/body mass index of HbSS and HbAA study participants

Variable	HbSS		HbAA	
	**R**	***P*-value**	**R**	***P*-value**
Age	-0.255	0.020	-0.425	<0.001*
BMI	0.035	0.733	-0.240	0.029

r = Pearson’s correlation coefficient, * Statistically significant

In Table 4, the HbSS phenotype status and age of participants were significant independent predictors of serum AMH levels (*p* <0.001) after univariate linear regression modelling.

**Table 4:** Regression analysis of the predictors of serum anti-Mullerian Hormone participants with HbSS and HbAA

Variables	Simple linear regression			Multiple linear regression		
	Coefficient (β)	95%Confidence interval	*P*-value	Adjusted Coefficient (adjusted β)	95% Confidence interval	*P*-value
**HbSS**	-4.24	-5.68 to -2.80	<0.001*	-4.42	-5.88 to -2.96	<0.001*
^**$**^**BMI**	-0.02	-0.07 to 0.03	0.457	-0.02	-0.06 to 0.03	0.436
**(Kg/m**^**2**^**)**						
Age	-0.19	-0.29 to -0.08	<0.001*	-0.18	-0.28 to -0.	<0.001*
(years)						

^$^ BMI: Body Mass Index, * statistically significant at *P*-value <0.05, Reference: HBAA

### Multivariate regression analysis

#### Haemoglobin (Hb) phenotype and serum AMH

After controlling for potential confounding variables (such as age, and BMI), we found that on the average the serum AMH level was about 4.42 ng/mL less among HbSS participants as compared to HbAA participants (adjusted β = - 4.42, 95% CI: -5.88 to -2.96, *P*-value <0.001).

#### Age and AMH

After controlling for confounding variables (such as Hb phenotype status and BMI), we found that for every yearly increase in age, the serum AMH level decreased by 0.18 ng/mL among participants (adjusted β = - 0.18, 95% CI: -0.28 to -0.08, *P*-value <0.001)

## Discussion

This study aimed to determine and compare the ovarian reserve among Nigerian women with HbSS and those with HbAA, using serum AMH as a marker, in order to document the impact of haemoglopinothies on the reproductive health of affected women. The exhaustive literature search suggest that this is the first and larger study to utilize serum AMH to evaluate ovarian reserve among HbSS women within the age range of 18 - 45 years in Nigeria and sub-Saharan Africa to the best of our knowledge.

This study found that the mean serum AMH level among women with HbSS was significantly lower than the mean serum AMH level among women with HbAA. This result is in agreement with the findings by Kopeika et al., in United Kingdom [19], that reported a mean AMH of 7.6 pmol/L (1.06 ng/mL) among HbSS participants as compared to a value of 13.4 pmol/L (1.88 ng/mL) among HbAA participants. The similarity in our study and that of Kopeika et al., may be explained by the fact that both studies recruited participants with similar characteristics and restricted the age limit of study participants to the reproductive age group. In contrast, Ibrahim and co-workers reported no significant differences in mean concentration of serum AMH between sickle cell disease participants and non-sickle cell disease participants [2]. In addition, the study by Ibrahim et al., recruited participants between the ages of 2 years and 38 years. The inclusion of the pre-pubertal participants could have significant impact on their findings, as serum AMH levels are low in children and adolescent girls [13].

This study revealed that a significantly higher proportion of HbSS participants had low serum AMH levels compared with age-matched HbAA participants. Similarly, the proportion of participants with high serum AMH value was also found to be higher in HbAA as compared to HbSS study participants. This may imply diminished ovarian reserve among HbSS study participants. The finding is similar to a previous study, which reported that the women with HbAA had a higher AMH levels compared to women with SCD [19].

Subgroup analyses of the relationship between serum AMH and age in HbSS and HbAA participants showed significant negative correlations between serum AMH levels and the age in both study groups. Our finding is consistent with the finding of negative correlation observed between the serum AMH and age of infertile women by Scheffer et al. [20] Our finding is also similar to the result of a large retrospective study consisting of 219,227 Asian Indian women aged 18-50 years which reported a negative correlation between the serum AMH levels and age [21].

In this study, there was a statistically significant difference in the mean BMI of the two groups of participants. The observed difference in BMI is likely due to the haemoglobinopathy and its long-term effect on growth and development. Although a negative correlation was observed between AMH and BMI in the HbAA, the study found no correlation between serum AMH level and BMI among the HbSS study participants. The latter is similar to findings of previous studies by Zahra et al. [22], and Buyuk et al. [23], which showed no significant linear relationship between BMI and serum AMH levels in women of reproductive- age group. The possible explanation for this finding may be the higher proportion (90.4% versus 60.0%)) of HbSS study participants to HbAA study participants who had low or normal BMI at enrollment.

All the participants from both groups in this study were of African race, but from different ethnic backgrounds in Nigeria. The predominant ethnic group of our participants was Yoruba. The strength of this study includes the cross-sectional analytical study design and the large population of women sickle cell anaemia within the reproductive age group.

This study has significant clinical implications. Firstly, the study provides baseline data on the potential impact of haemoglobinopathies on the ovarian reserve of women with sickle cell anaemia within the age range of 18 - 45 years in Nigeria, a country with the highest burden of the disease. Secondly, the findings from this study suggest a need to monitor the ovarian reserve of women with sickle cell anaemia and assess the impact of the declining ovarian reserve on their reproductive functions. Lastly, this study highlights the need for further longitudinal studies on the cause-effect relationship between haemoglobinopathies (sickle cell disease and sickle cell trait) and ovarian reserve. Findings from this study and future longitudinal studies can greatly influence clinical practice and counselling, especially regarding fertility preservation options for women with sickle cell disease who wish to delay childbearing.

## Limitations of the study

Firstly, the study was hospital-based and the findings may not be a true representation of the general population but the study has enough power to make generalization. Secondly, the possibility of primary ovarian insufficiency/premature ovarian failure cannot be completely excluded as serum oestrogen and serum pituitary gonadotropins (FSH and LH) were not assayed. However, all study participants were still having regular menstruation at the time of enrolment into the study. In addition, the antral follicular count and inhibin B were not measured and the subjects were not fully evaluated for polycystic ovarian syndrome, a disorder associated with abnormally high serum AMH. However, attempt was made at recruitment to exclude women with menstrual disorders, using the screening form.

## **C**onclusion

Women living with sickle cell anaemia may be at risk of diminished ovarian reserve and by extension, reduced reproductive ability.

## Recommendation

Larger longitudinal studies of serum AMH and other markers of ovarian reserve are necessary in women with sickle cell disease, to corroborate our findings. Women with sickle cell anaemia within the reproductive age group may need to have their ovarian reserve tested regularly.

## Data Availability

The data that support the findings of this study are available from SRG but restrictions apply to the availability of these data, which were used under license for the current study, and so are not publicly available. Data are however available from the authors upon reasonable request and with permission of CCM.

## Abbreviations

AFC: Antral follicle count
AMH: Anti- Mullerian Hormones
AOR: Assessment of Ovarian Reserve
BMI: Body mass index
DOR: Diminished ovarian reserve
E2: Basal oestradiol
ED: Erectile dysfunction
EDTA: Ethylene diamine tetraacetic acid
ELISA: Enzyme-linked immunosorbent assay
FSH: Basal follicle stimulating hormone
GnRH: Gonadotropin- releasing hormones
Hb A: Adult Haemoglobin
Hb S: Sickle Haemoglobin
HbAA: Haemoglobin AA
HbSC: Haemoglobin SC
HbSD: Haemoglobin SD Punjab disease
HbSE: Haemoglobin SE
HbSO: Haemoglobin SO Arab disease
HbSS: Haemoglobin SS Phenotype
HbSβ+: Haemoglobin S-beta-Thalassemia plus
HbSβ0: Haemoglobin S-beta-thalassemia zero
Kg: Kilograms
LH: Luteinizing hormone
LUTH: Lagos University Teaching Hospital
ML: Milliliters
ng/ml: Nanograms per milliliter
OR: Ovarian Reserve
pg/L: Picograms per liter
SCA: Sickle cell anaemia
SCD: Sickle cell disease
SD: Standard Deviation
SPSS: Statistical package for the social sciences
SST: Serum Separator Tubes
TGF-β: Transforming growth factor beta
U/L: International unit per litre
UK: United Kingdom
US: United States
WHO: World Health Organization
